# Supplementary material for: Stress-induced dysfunction of neurovascular astrocytes in the prefrontal cortex contributes to sex-dependent deficits in cognition and behavior
Source: Mol Psychiatry. 2025 Apr 4;30(9):4128–41. doi: 10.1038/s41380-025-02993-3 (PMC12339369; doi:10.1038/s41380-025-02993-3)
Supplement: Supplementary file 1 — Supplemental Figures 1-3 [file 41380_2025_2993_MOESM1_ESM.pdf]

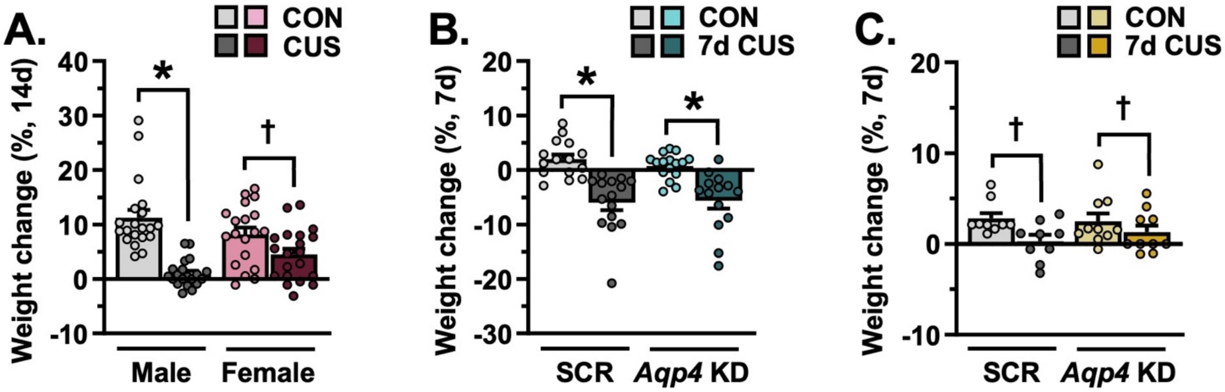

Supplementary Figure 1. Chronic stress reduces animal weight gain. A) Weight change in mice subjected to CUS or left unstressed (14-days). Stress differentially reduced weight gain in male and female mice ($F_{(1,75)}$=8.148, *p*=0.005). Planned comparisons indicate a significant reduction in weight gain in stressed males (*p*<0.0001) and a trend toward this in stressed females (*p*=0.089). **B-C)** Weight change in male (B) and female (C) mice subjected to sub-CUS or left unstressed (7-days). Exposure to sub-CUS reduced weight gain in both sexes, regardless of *Aqp4* knockdown in the PFC (Males: $F_{(1,56)}$=41.98 *p*<0.0001; Females: $F_{(1,34)}$=6.154, *p*=0.018). Bars represent mean ± S.E.M. Graphed points indicate data derived from individual animals. † p<0.05 main effect (specific group difference not detected by planned comparison). * p<0.05 planned comparison indicated (Sidak's test following a significant main effect or interaction).

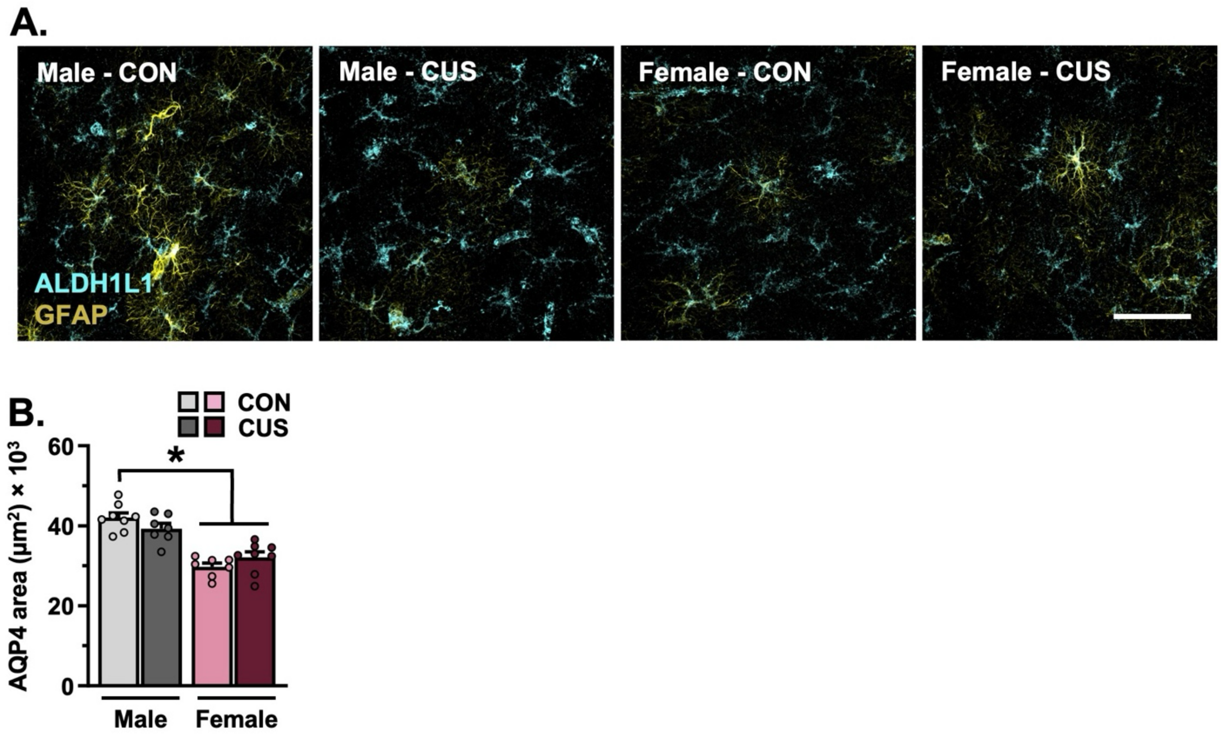

Supplementary Figure 2. Sex differences in the total area of astrocyte AQP4 coverage in the prefrontal cortex. A) Representative images of ALDH1L (Cyan) and GFAP (yellow) immunohistology in the PFC (20× + zoom, scale bar = 50 μm. **B)** Total area of AQP4+ material in the PFC. Bars represent mean ± S.E.M. Graphed points indicate data derived from individual animals. * $p<0.05$ planned comparison indicated (Sidak's test following a significant main effect or interaction).

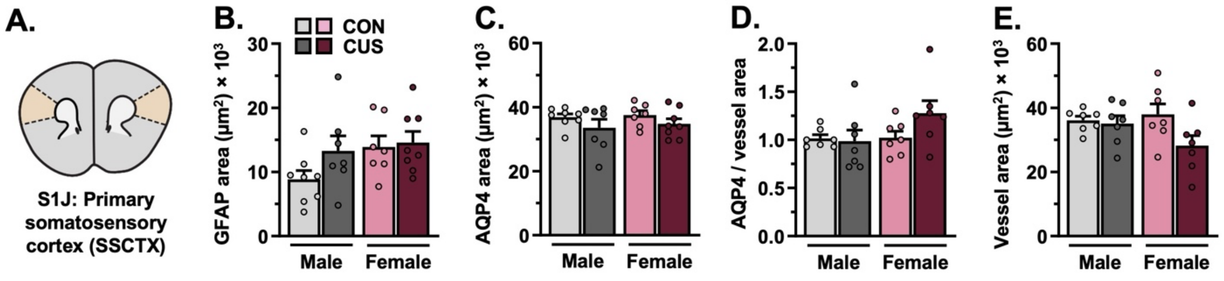

Supplementary Figure 3. Analysis of astrocyte coverage and blood vessel area in the somatosensory cortex. A) Schematic of the S1J subregion of the SSCTX. Analysis of this region occurred in the same animals as presented in Figure 3. **B)** Area of GFAP+ material in the SSCTX. **C)** Total area of AQP4+ material in the SSCTX. **D)** Area of astrocyte AQP4+ material relative to vessel area. **E)** Area of tomato lectin+ vessels in the SSCTX. Bars represent mean ± S.E.M. Graphed points indicate data derived from individual animals.
